# Study of FOXO1-interacting proteins using TurboID-based proximity labeling technology

**DOI:** 10.1186/s12864-023-09238-z

**Published:** 2023-03-24

**Authors:** Yanting Su, Yuanyuan Guo, Jieyu Guo, Ting Zeng, Ting Wang, Wu Liu

**Affiliations:** 1 Medicine Research Institute, Hubei Key Laboratory of Diabetes and Angiopathy, Xianning Medical College, Hubei University of Science and Technology, Xianning, Hubei 437000 China; 2 School of Basic Medical Sciences, Xianning Medical College, Hubei University of Science and Technology, Xianning, Hubei 437000 China; 3 School of Pharmacy, Xianning Medical College, Hubei University of Science and Technology, Xianning, Hubei 437000 China; 4 Department of Pediatric Neurology, Maternal and Child Health Hospital of Hubei Province, Wuhan, Hubei 430000 China

**Keywords:** TurboID, FOXO1, Biotin labeling, U251 cells, hnRNPK

## Abstract

**Background:**

Protein‒protein interactions (PPIs) are the foundation of the life activities of cells. TurboID is a biotin ligase with higher catalytic efficiency than BioID or APEX that reduces the required labeling time from 18 h to 10 min. Since many proteins participate in binding and catalytic events that are very short-lived, it is theoretically possible to find relatively novel binding proteins using the TurboID technique. Cell proliferation, apoptosis, autophagy, oxidative stress and metabolic disorders underlie many diseases, and forkhead box transcription factor 1 (FOXO1) plays a key role in these physiological and pathological processes.

**Results:**

The FOXO1-TurboID fusion gene was transfected into U251 astrocytes, and a cell line stably expressing FOXO1 was constructed. While constructing the FOXO1 overexpression plasmid, we also added the gene sequence of TurboID to perform biotin labeling experiments in the successfully fabricated cell line to look for FOXO1 reciprocal proteins. Label-free mass spectrometry analysis was performed, and 325 interacting proteins were found. A total of 176 proteins were identified in the FOXO1 overexpression group, and 227 proteins were identified in the Lipopolysaccharide -treated group (Lipopolysaccharide, LPS). Wild-type U251 cells were used to exclude interference from nonspecific binding. The FOXO1-interacting proteins hnRNPK and RBM14 were selected for immunoprecipitation and immunofluorescence verification.

**Conclusion:**

The TurboID technique was used to select the FOXO1-interacting proteins, and after removing the proteins identified in the blank group, a large number of interacting proteins were found in both positive groups. This study lays a foundation for further study of the function of FOXO1 and the regulatory network in which it is involved.

**Supplementary Information:**

The online version contains supplementary material available at 10.1186/s12864-023-09238-z.

## Background

The forkhead transcription factor (FOXO) family of proteins can inhibit tumor proliferation, regulate energy metabolism, induce cell responses, and contribute to the regulation of human antiaging due to their widespread presence in various [1–3]. Many stimuli can induce changes in FOXO activity, such as insulin, insulin-like growth factor-1 (IGF-1), cytokines and oxidative [4]. After in-depth study of the FOXO family, forkhead box transcription factor 1 (FOXO1) has been considered a representative member because it plays a key regulatory role in many transcription [5]. A large number of studies have shown that FOXO1, regarded as an important transcription factor due to its complex activities, regulates many targets, such as genes involved in apoptosis and autophagy, antioxidant enzymes, cell cycle arrest genes, and metabolic and immune regulatory [6, 7]. However, the mechanism of FOXO1 in the occurrence and development of many diseases is still unclear and even contradictory. Therefore, in-depth study of the FOXO1 signaling pathway is of great significance for the development of targeted drugs for a variety of [8].

As people’s quality of life has improved in recent years, diabetes has become a familiar disease, with insulin resistance and β-cell function impairment being the hallmarks of type 2 diabetes mellitus (T2DM). The concept of insulin resistance emerged in the 1980 and 1990 s and has been recognized as the basic pathological state of T2DM, while β-cell exhaustion has received less attention. Studies have shown that far fewer individuals have T2DM than those that have insulin resistance alone, suggesting that insulin resistance does not necessarily lead to T2DM unless it is also accompanied by pancreatic β-cell [8–11]. Healthy β-cells can increase their numbers and functional output to compensate for the effects of insulin [12]. In T2DM, β-cell dysfunction can be induced in many ways, including oxidative [13], endoplasmic reticulum [14], hypoxic [15] and the expression of [16], which lead to apoptosis, restricted proliferation, uncontrolled autophagy, the dedifferentiation of β-cells and other adverse consequences. Studies have shown that FOXO1 is involved in the above mechanisms and exerts corresponding functions. Although FOXO1 inhibits β-cell replication and neogenesis, it is required to maintain the functions and characteristics of β-cells during times of increased metabolic [17]. Thus, FOXO1 is an indispensable factor in diabetes research. In view of the important role of FOXO1 in T2DM, we aimed to construct a cell line stably overexpressing the FOXO1 gene to identify the proteins with which it interacts to facilitate the study of its mechanism of action in the future.

Protein‒protein interactions (PPIs) are the foundation of the life activities of [18]. Protein proximity labeling technologies, such as BioID and APEX, have gradually been applied to study of [19, 20]. Since the interactions between proteins rely mostly on hydrogen bonds, salt bridges and hydrophobic interactions and their spatial distance from each other is very short, it is generally considered that interacting proteins must be adjacent to each [21]. TurboID is a protein with a higher catalytic efficiency than BioID or APEX, and it reduces the labeling time from 18 h to 10 min. Since many proteins exert binding and catalytic effects that are very short-lived, it is theoretically possible to find relatively novel binding proteins using the TurboID [22]. Here, we utilize the efficiency of TurboID to find proteins that interact with FOXO1.

To conduct in-depth research on FOXO1, this paper used lentivirus infection to transfect the FOXO1 gene into U251 astrocytes to construct a stable cell line that expresses FOXO1. While constructing the FOXO1 overexpression plasmid, we also included the gene sequence of TurboID. Therefore, we can conduct biotin labeling experiments on the successfully constructed cell line and design an experimental group for LPS treatment. A large number of proteins that interact with FOXO1 were found by silver staining, which lays a foundation for further study of the function of FOXO1 and the regulatory network in which it is involved.

## Results

### Construction of overexpression plasmid and TurboID labeling flowchart

**Fig. 1:**
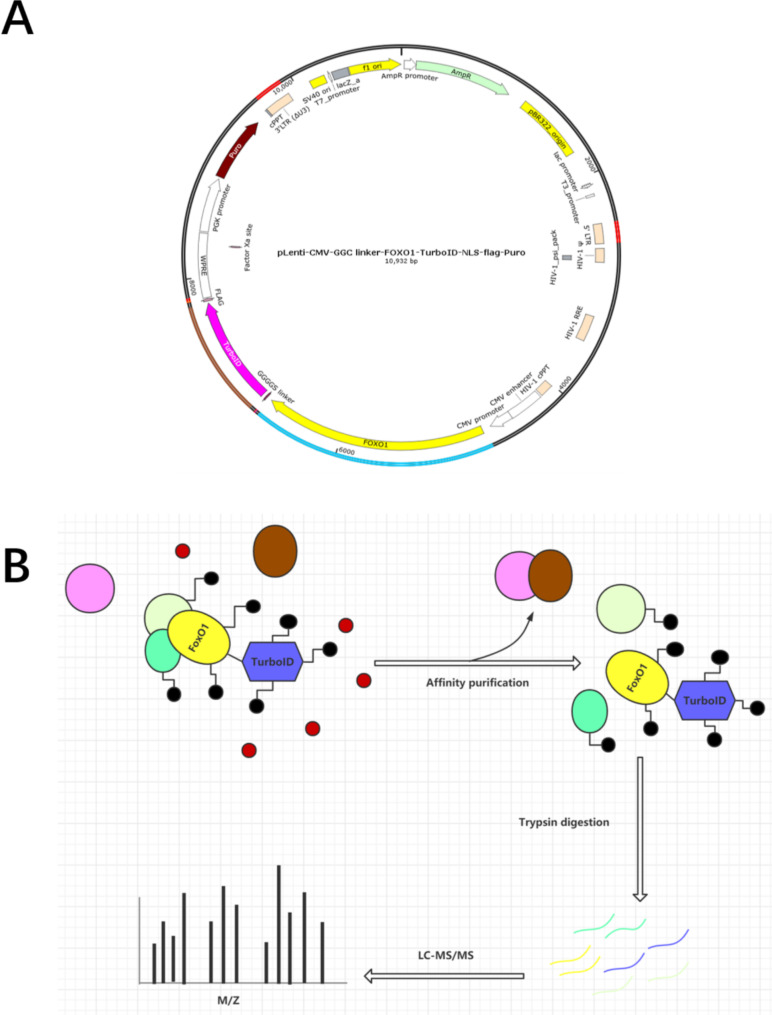
Construction of the overexpression plasmid and TurboID labeling flowchart. (A) Diagram of functional elements of the FOXO1-TurboID overexpression plasmid. **(B)** Biotin marker experimental workflow

The composition of the FOXO1-TurboID overexpression plasmid is shown in Fig. 1A, including the target gene FOXO1, biotin marker enzyme TurboID, nuclear localization signal (NLS) and tag protein Flag, as well as two different resistance screening markers, AmpR and puro, where AmpR was used to screen for positive clones in *E. coli*, and puro was used to screen for stably transfected cells. Figure 1B is a simple illustration of our biotin experiment. First, the successfully and stably transfected FOXO1-U251 cells were placed in an environment with a suitable concentration of biotin so that free biotin could fully enter the cells, and adjacent proteins were covalently labeled with biotin by TurboID. After the protein affinity purification experiment, proteins that were not labeled with biotin were excluded, the labeled proteins were selected for analysis by label-free quantitative mass spectrometry analysis.

### Construction and validation of the stably transfected cell line

**Fig. 2:**
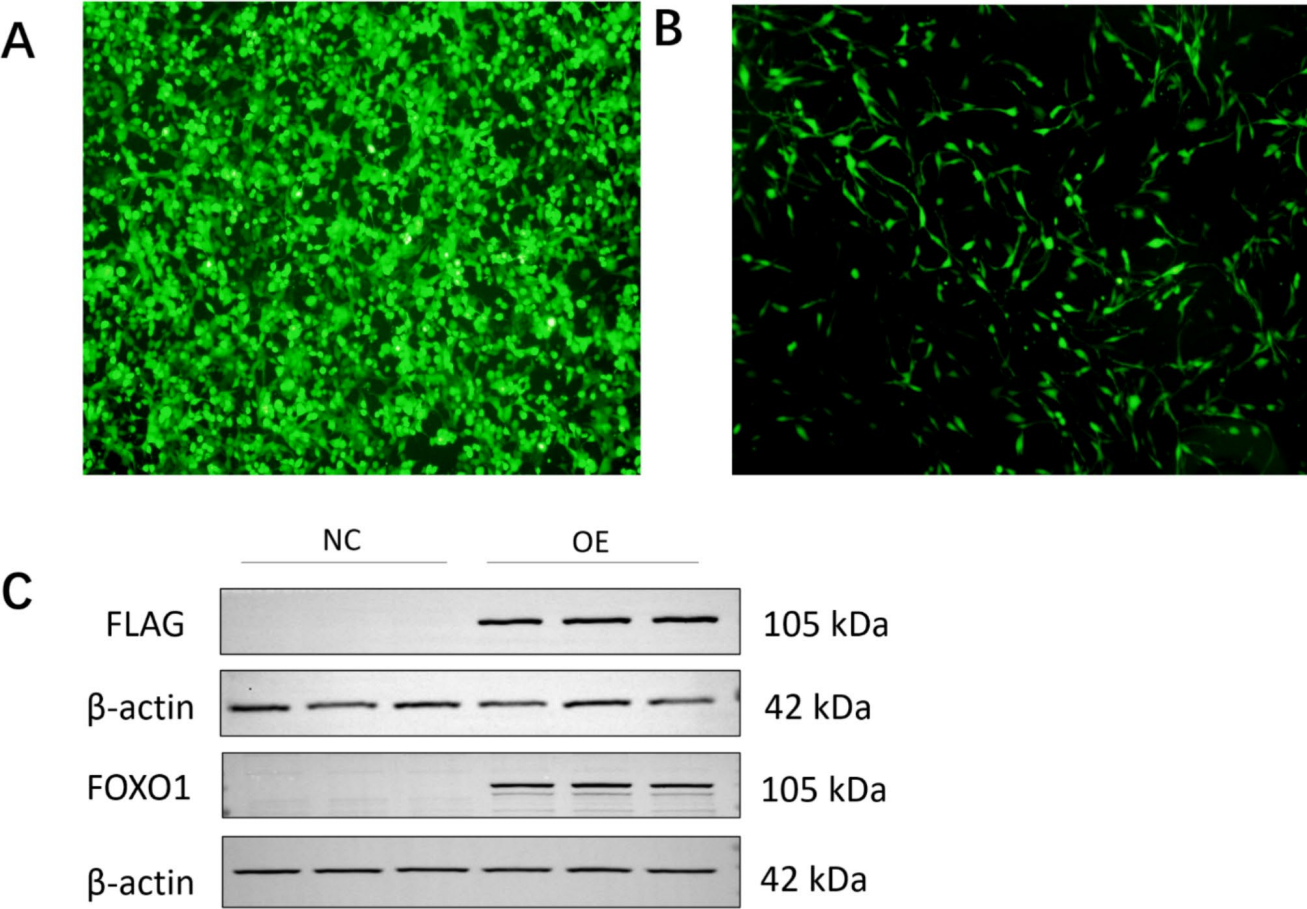
Validation of cell line stable transfection. (A) Fluorescence image of 293T cells highly expressing pLenti-CMV-EGFP. (B) Fluorescence image of U251 cells highly expressing pLenti-CMV-EGFP. (C) Validation of FOXO1 protein expression. NC, U251 cells; OE, cells with stable overexpression of FOXO1-TurboID

In Figs. 2A and 293T cells were cotransfected with PSPAXZ, PMD2.G and pLenti-CMV-EGFP overexpression plasmids. After 48 h of infection, intense fluorescence was observed under a fluorescence microscope, which indicated that the cells had been successfully transfected with the pLenti-CMV-EGFP gene. There were no problems in the transfection system, and so the same batch of the FOXO1-TurboID cotransfection system should proceed smoothly, and the supernatant virus solution after transfection can be collected. Figure 2B shows the U251 cells observed under a fluorescence microscope 48 h after lentivirus infection. The appearance of fluorescence also confirms that the pLenti-CMV-EGFP gene was successfully transfected into the U251 cells, making the probability of FOXO1-TurboID transfection high.

The cells overexpressing the FOXO1 gene were treated with Flag antibody for Western blotting and a clear band appeared at the protein molecular weight of approximately 105 kDa (FOXO1 protein molecular weight 69.6 kDa, TurboID protein molecular weight 35 kDa; the size of Flag is almost negligible) (Fig. 2C). This region was blank in the U251 cell control group, indicating that a stable cell line was successfully constructed. The results obtained with the FOXO1 antibody were similar.

### Biotin labeling and identification by silver staining

**Fig. 3:**
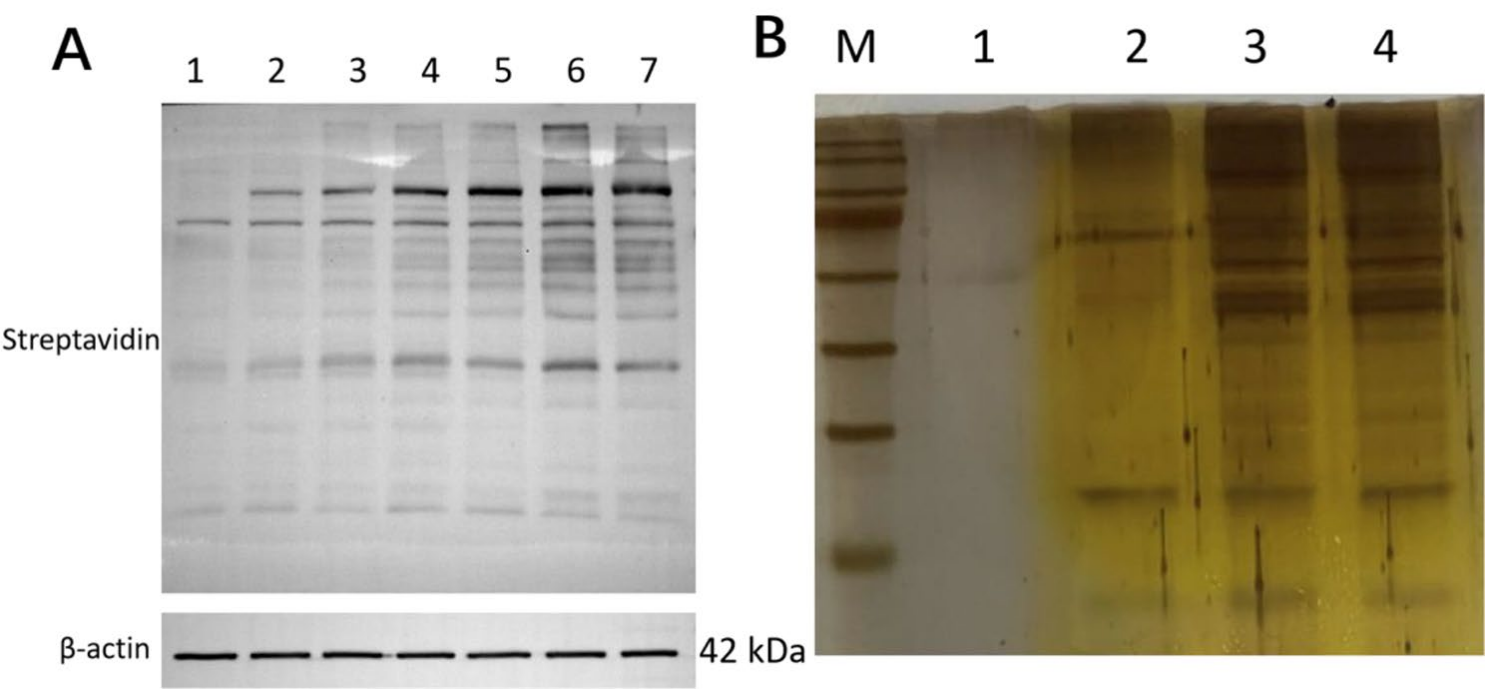
Biotin labeling and silver staining. (A) Biotin labeling was assessed at different time points. 1, 0 min; 2, 10 min; 3, 1 h; 4, 3 h; 5, 6 h; 6, 12 h; 7, 24 h. (B) Protein identification by silver staining. M, protein ladder; 1, Blank Strip; 2, EGFP-U251 cells; 3, FOXO1-U251 cells; 3, LPS-treated FOXO1-U251 cells

Before performing the biotin labeling experiments, we determined the time needed for biotin labeling. Figure 3 A shows that 12 h was the appropriate time. Therefore, 500 µmol/L biotin was used to treat the stably transfected FOXO1-TurboID cells for 12 h.

After the three groups of cells were treated with biotin for 12 h for labeling, the biotin-labeled protein was pulled down with magnetic beads and then identified by silver staining. The significant difference between the cell line stably expressing FOXO1 and control EGFP-U251 cells is shown in Fig. 3B, which proves that a large number of proteins that interact with the target protein FOXO1 had been pulled down. The difference in expression between FOXO1-U251 cells and LPS-treated FOXO1-U251 cells needs further mass spectrometry analysis.

### Mass spectrometry data analysis

By analyzing the mass spectrometry data, a total of 325 interacting proteins were collected by the proximity labeling technique. One hundred proteins were identified in the U251 blank group, and 240 proteins were identified in the FOXO1 overexpression group. After excluding 64 proteins that were also identified in the blank group, 176 interacting proteins remained (see the Additional file 1 for specific information on these proteins). Additionally, 289 proteins were identified in the LPS-treated group, and after excluding the 62 proteins that were also identified in the blank group, 227 interacting proteins remained (see the Additional file 1 for specific information on these proteins).

**Fig. 4:**
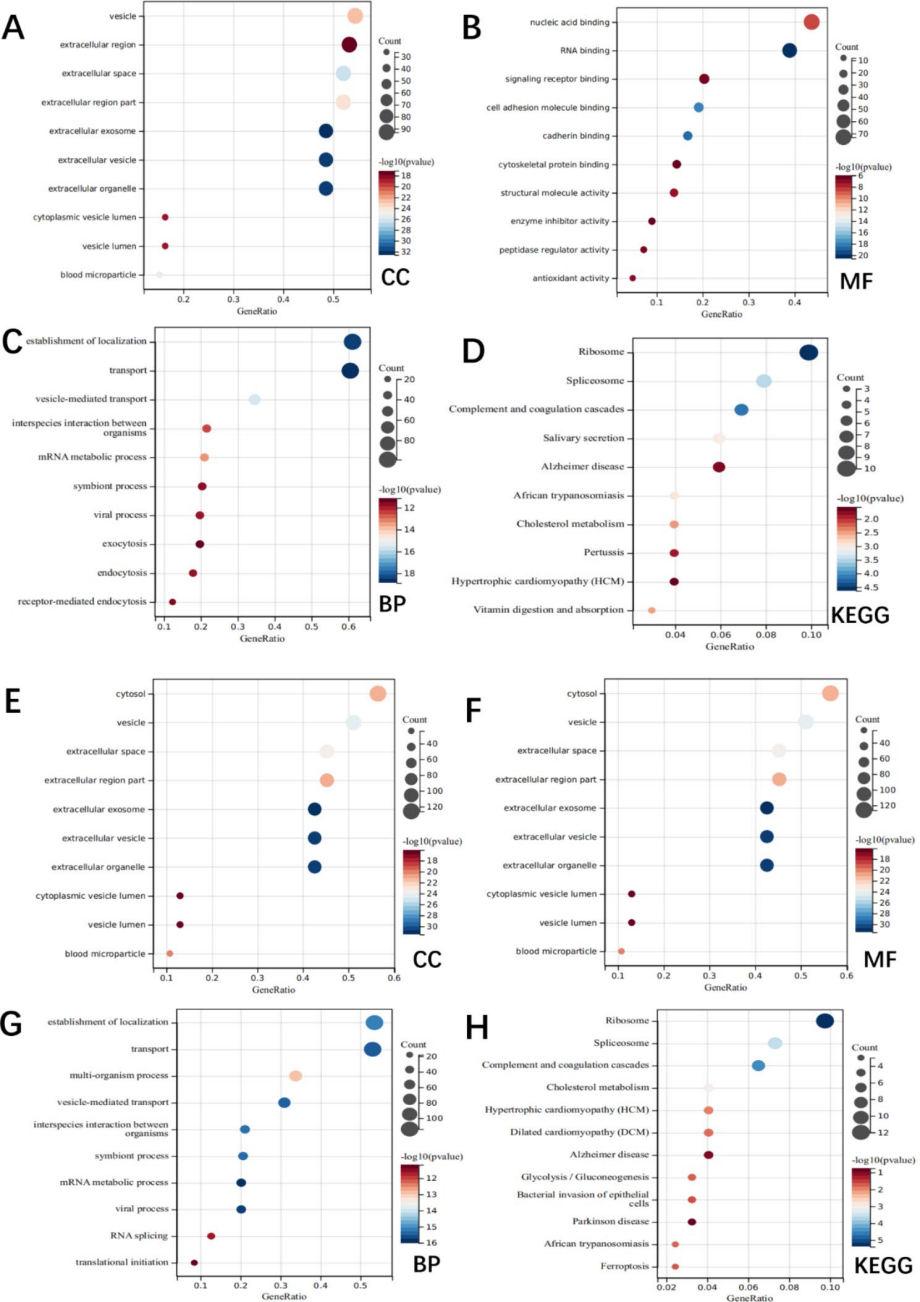
Analysis of FOXO1 proximates. (A) Enriched proximate proteins of the FOXO1 group based on cellular components. (B) Enriched proximate proteins of the FOXO1 group based on molecular function. (C) Enriched proximate proteins of the FOXO1 group based on biological process. (D) Enriched proximate proteins of the FOXO1 group based on KEGG analysis. (E) Enriched proximate proteins of the LPS-treated group based on cellular components. (F) Enriched proximate proteins of the LPS-treated group based on molecular function. (G) Enriched proximate proteins of the LPS-treated group based on biological process. (H) Enriched proximate proteins of the LPS-treated group based on KEGG analysis

We used the online analysis tool Sangerbox 3.0 for GO and KEGG analyses (Sangerbox 3.0, http://vip.sangerbox.com/home.html) [23–25]. First, we selected 176 interacting proteins from the FOXO1 overexpression group. In the analysis of cellular components, proteins were found to be enriched in vesicle, extracellular region, extracellular space, extracellular region part, extracellular exosome, etc. (Fig. 4A). In the analysis of molecular function, proteins were found to be enriched in nucleic acid binding, RNA binding, signaling receptor binding, cell adhesion molecule binding, cadherin binding, etc. (Fig. 4B). In addition, several significantly enriched proteins were identified from the analysis of biological processes, including establishment of localization, transport, vesicle-mediated transport, interspecies interaction between organisms, mRNA metabolic process, etc. (Fig. 4C). After KEGG analysis, proteins were found to be enriched in the following pathways: ribosome, spliceosome, complement and coagulation cascades, salivary secretion, Alzheimer’s disease, African trypanosomiasis, cholesterol metabolism, pertussis, etc. (Fig. 4D).

Next, we selected 227 interacting proteins from the LPS-treated group for analysis. In the analysis of cellular components, the following protein enrichments were found, including cytosol, vesicle, extracellular space, extracellular region part, extracellular exosome, etc. (Fig. 4E). In the analysis of molecular function, the search for nucleic acid binding, RNA binding, cell adhesion molecule binding, cadherin binding, protein-containing complex binding, etc. (Fig. 4F). In addition, several significantly enriched regions were identified in the analysis of biological processes, including establishment of localization, transport, multiorganism process, vesicle-mediated transport, interspecies interaction between organisms, etc. (Fig. 4G). After KEGG analysis, proteins were found to be enriched in the following pathways: ribosome, spliceosome, complement and coagulation cascades, cholesterol metabolism, hypertrophic cardiomyopathy (HCM), dilated cardiomyopathy (DCM), Alzheimer’s disease, glycolysis/gluconeogenesis, Parkinson’s disease, ferroptosis, etc. (Fig. 4H).

### Validation of interacting proteins

**Fig. 5:**
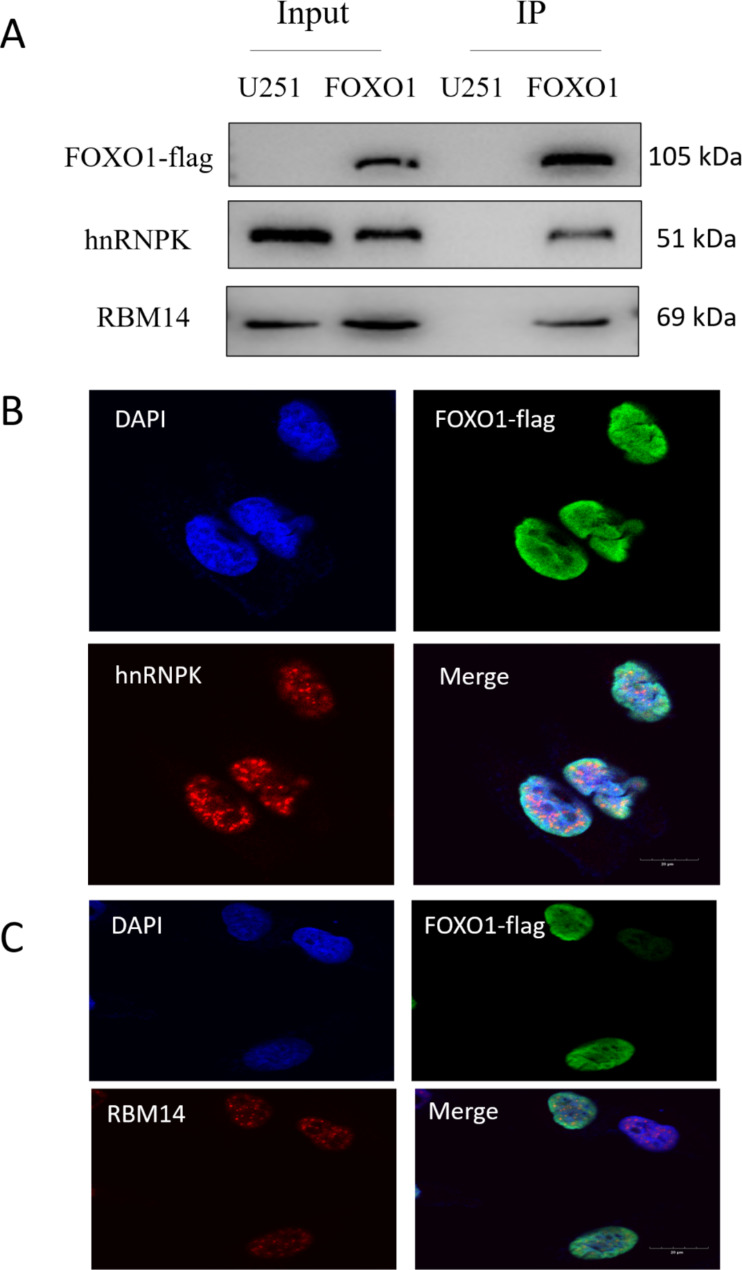
Validation of the interacting proteins. (A) Western blotting bands after immunoprecipitation. (B) Immunofluorescence image of the interaction between FOXO1 and hnRNPK in FOXO1-U251 cells. (C) Immunofluorescence image of the interaction between FOXO1 and RBM14 in FOXO1-U251 cells

The mass spectrometry data indicated that many hnRNP family members are FOXO1-interacting proteins, including hnRNPF, hnRNPD, hnRNPA1, hnRNPH1, hnRNPA2B1, hnRNPH3, hnRNPA3, and hnRNPK. Therefore, hnRNPK was selected for protein interaction verification. In addition, RBM14, a nuclear receptor coactivator, was selected. Cells were collected and subjected to immunoprecipitation experiments. The Flag affinity gel adsorbs the target protein with the Flag tag, so it can adsorb FOXO1-TurboID-Flag fusion proteins. Figure 5 A shows that FOXO1 can specifically bind to hnRNPK and RBM14. Therefore, these samples after immunoprecipitation could show bands for the hnRNPK and RBM14 antibodies.

We used immunofluorescence techniques to verify the interaction between FOXO1 and hnRNPK. The immunofluorescence staining results were observed by confocal microscopy. In Figs. 4 and 5B’,6-diaminyl-2-phenylindoles (DAPI) emits blue fluorescence and shows the morphology of the nucleus, the fluorescent secondary antibody fluorescein isothiocyanate (FITC, green fluorescence) marks the FOXO1 gene, and the fluorescent secondary antibody Sulfo-Cyanine3 (Cy3, red fluorescence) indicates hnRNPK. The merged image has many yellow spots, which may indicate a large number of interacting FOXO1 and hnRNPK proteins. Similarly, in Fig. 5C, DAPI emits blue fluorescence, showing the morphology of the nucleus, the fluorescent secondary antibody FITC (green) marks the FOXO1 gene, and the fluorescent secondary antibody Cy3 (red) indicates RBM14. The combined image has many yellow spots, which may indicate a large number of interacting FOXO1 and RBM14 proteins. However, because the expression of FOXO1 is too high and diffuses throughout the nucleus, the combination result of co-localization may be questionable. Relevant experiments need further verification. Although the confocal image data is doubtful, we still believe that FOXO1 interacts with two proteins through immunoprecipitation experiments.

## Discussion

In recent years, people have paid increasing attention to their health, and research on health preservation and antiaging has received more interest. The FOXO family genes are recognized as a class of longevity [26]. FOXO-regulated genes mainly include those that regulate the cell cycle and cellular death, autophagy, metabolism, and antioxidative processes. FOXO, as a switch that regulates a large class of functional genes, is an important research object in medical biology and life science [27, 28]. Cell lines that stably express FOXO can provide a reliable platform to study this family. The overexpression of genes in cells can be either transient or stable. Transient overexpression is relatively limited in scope and [29]. For example, transiently overexpressed RNA can only produce interference for a certain period of time in experiments. Transient transfer will introduce unpredictable copy number expression (while transient expression tends to be high and very unstable), resulting in inaccurate experimental results due to artificial factors. However, stable gene interference effects can be achieved by constructing stable cell [30]. For accurate and stable cell experiments, it is necessary to construct stably transfected cell lines.

Since most of the proteins encoded by human genes perform their functions when in [31], it is necessary to identify protein interactions to deeply analyze the structure and function of cells and study their molecular basis. However, most protein interactions are weak and transient, and overcoming these disadvantages has been a major difficulty. In recent years, Branon et al. introduced TurboID, a novel enzyme that biotinylates adjacent proteins, which combines the simplicity and nontoxicity of BioID with the high catalytic efficiency of [32]. This technique uses biotinylated enzymes to identify adjacent proteins and then enriches the biotin-labeled proteins with avidin magnetic beads for identification by mass spectrometry. Overall, using TurboID significantly improves the efficiency and greatly reduces the labeling time required compared with the original biotin labeling [22].

Lentiviral transfection, featuring high efficiency, stability and precision, has become a common tool for research in cellular and molecular [33]. Lentiviruses not only permit the continuous expression of the target protein in cells but are also puromycin-resistant to allow for the rapid selection of cell lines. In this study, we used a lentivirus to construct FOXO1-TurboID and EGFP-TurboID overexpression vectors, and EGFP-TurboID cells showed green fluorescence, indicating that the active lentiviral particles had been successfully delivered. FOXO1-TurboID expression was detected at the protein level, and a cell line that continuously expressed the FOXO1-TurboID gene was obtained. Next, we performed biotin labeling experiments on wild-type U251 cells and U251 cells with stable overexpression of FOXO1-TurboID and treated the latter cells with LPS. Subsequently, after biotin affinity purification and silver staining, a large number of proteins that interact with FOXO1 were directly observed after excluding the endogenous biotin-interacting proteins found in the false-positive control sample.

In this experiment, a total of 325 interacting proteins were collected by the proximity labeling technique. One hundred proteins were identified in the U251 blank group, and 240 proteins were identified in the FOXO1 overexpression group. After excluding the 64 proteins that were also identified in the blank group, 176 interacting proteins remained. Moreover, 289 proteins were identified in the LPS-treated group, and after excluding the 62 proteins that were also identified in the blank group, 227 interacting proteins remained. We then performed GO analysis and KEGG analysis. All of the above analyses are in line with the characteristics of FOXO1 as a transcription factor. In the LPS-treated group, several interesting pathways were enriched according to KEGG analysis, including hypertrophic cardiomyopathy (HCM), dilated cardiomyopathy (DCM), Alzheimer’s disease, glycolysis/gluconeogenesis, Parkinson’s disease, and ferroptosis. These pathways deserve further study.

The mass spectrometry data indicated that many hnRNP family members are FOXO1-interacting proteins, including hnRNPF, hnRNPD, hnRNPA1, hnRNPH1, hnRNPA2B1, hnRNPH3, hnRNPA3, and hnRNPK. Therefore, hnRNPK was selected for protein interaction verification. In addition, RBM14, a nuclear receptor coactivator, was selected. FOXO1 interacts with hnRNPK and RBM14, as verified by immunoprecipitation and confocal experiments. hnRNPK is an important regulatory protein in the nuclear heterogeneous ribonucleoprotein (hnRNP) [34] that is widely expressed in mammalian cells and distributed in the nucleus, cytoplasm, mitochondria and cell [35]. As the center of many biological pathways, hnRNPK is crucial for gene expression regulation, cell signal transduction, DNA repair and telomere [36]. A study found that hnRNPK is a conserved DNA/RNA binding protein that has a high expression level in tumor tissues and is closely related to the prognosis of malignant [37]. In addition, Gallardo et al. found that the reduced survival rate of mouse embryos with hnRNPK knockout suggests that this protein may play a key role in the development of newborn [38]. FOXO1, an important transcription factor, is the main target of insulin signal transduction and regulates metabolic homeostasis during oxidative stress. We believe that the interaction between FOXO1 and hnRNPK may be involved in the regulation of the expression of a large group of genes and play certain roles in cancer and diabetes. RNA binding motif protein 14 (RBM14), also known as PSP2 or quasi dot protein, is an RNA binding protein belonging to the RBM (RNA binding motif) protein [39]. RBM 14 has two RNA recognition motifs (RRMs) and a prion-like domain (PLD) at the N-terminus. Because it can interact with RNA and proteins, it can play a variety of roles in eukaryotic cells, such as participating in transcriptional activation, inducing chromosome separation, helping with DNA repair and cell [40]. Studies have shown that the RBM protein family is crucial in mesodermal development, and RBM14 plays a major role in the development of the heart. When RBM14 is absent, a variety of embryonic defects may occur, such as cardiac abnormalities and heart [41]. RBM14 is also a potential factor affecting oocyte quality and meiotic maturation of [42]. We believe that the interaction between FOXO1 and RBM 14 may regulate embryonic development and participate in DNA repair and cell differentiation processes.

Although our study identified a large number of FOXO1-interacting proteins, the biological functions of such interactions need to be further confirmed. I hope our study will provide a reference for FOXO1-related disease research.

## Conclusion

In this study, label-free mass spectrometry analysis was performed to identify 325 interacting proteins. A total of 176 proteins were identified in the FOXO1 overexpression group, and 227 proteins were identified in the LPS-treated group. These numbers exclude the interfering proteins identified in the U251 blank group. Finally, the interacting proteins hnRNPK and RBM14 were selected for immunoprecipitation and immunofluorescence verification studies.

## Materials and methods

### Materials

Monoclonal antibodies [FLAG (ab205606), hnRNP K (ab134060), and RBM14 (ab70636)] were purchased from Abcam. FOXO1 (a2934) and β-actin (ac006) were purchased from ABclonal. Lipopolysaccharide (LPS, L8880) was purchased from Beijing Solarbio Technology Co., Ltd. PVDF membranes were purchased from Millipore. Dynabead™ MyOne™ streptavidin C1 (65,001), biotin (B20656) and liposomal transfection reagent (Lipofectamine 2000, 11,668,500) were obtained from Thermo Fisher. RIPA buffer (G2002-100ML), DAIP buffer (G1012-100ML), and anti-fluorescence quenching sealing agent (G1401-25ML) were purchased from Wuhan Servicebio Technology Co., Ltd. Anti-DYKDDDDK (Flag) Affinity Gel (20585ES03), Lentivirus Concentration Solution (41101ES50) and a protein silver staining kit (36244ES30) were purchased from Yisheng Biotechnology (Shanghai) Co., Ltd. A DNA product purification kit (DP204) and a high purity plasmid small amount extraction kit (DP104) were purchased from Tiangen Biotech (Beijing) Co., Ltd. All of the inorganic salts came from Sinopharm.

### Cells lines

HEK 293T and U251 cells were obtained from ATCC (Manassas, USA). All cells were grown in Dulbecco’s Modified Eagle’s Medium (Gibco, USA) supplemented with 10% fetal bovine serum (Gibco, USA). All cells were cultured in a 5% CO_2_-humidified atmosphere at 37 °C.

### Construction of the plasmids

The TurboID gene sequence was obtained from Dr. Bo Xu (Wuhan University) and it replaced EGFP for integration into the pLenti-CMV-EGFP plasmid to generate pLenti-CMV-TurboID. The FOXO1 gene was amplified with human cDNA as a template (primer, FOXO1-f: atagaagacaccaccacacttagaatggccgaggcgcctcag; FOXO1-r: ggggggtatacgtataccgcctg acaccagctatgtc). pLenti-CMV-TurboID used restriction sites Xba I and BamH I, and the FOXO1 fragment was recombined into the linearized pLenti-CMV-TurboID plasmid. The positive clones were screened by transformation. The extracted plasmid was identified by enzyme digestion and sent to a company for sequencing. The successfully sequenced plasmid was named FOXO1-TurboID (Addgene ID: 194,712).

### Lentivirus packaging of the plasmid

293T cells were plated in two 10 cm cell culture dishes and transfected when the cells reached a density of 80-90%. For transfection, serum-free medium was used to dilute the plasmids. One 1.5 mL EP tube contained dilutions of the PSPAXZ, PMD2.G and FOXO1-TurboID plasmids, and another EP tube contained dilutions of the PSPAXZ, PMD2.G and pLenti-CMV-EGFP plasmids. Later, an appropriate amount of liposomal transfection reagent (approximately 60 µl in a 10 cm dish) was diluted with serum-free medium and incubated at room temperature for 5 min. Then, the diluted DNA plasmid and liposome transfection reagent were mixed in slowly and evenly, and the samples were placed at room temperature for 20 min. Next, the prepared DNA–liposome complex was added to a 10 cm Petri dish with media exchange and cultured overnight at 37 °C. After exchanging the media again and further culture for 48–72 h, the medium was collected. The culture medium was removed with a 10 mL syringe and filtered through a 0.45 mm filter membrane. The filtrate was mixed with the concentrated lentivirus reagent in an appropriate proportion (reagent:culture medium = 1:4). Finally, the supernatant was centrifuged, fresh culture medium was mixed in for precipitation, and the virus was aliquoted into several 1.5 mL EP tubes.

### Construction and validation of the FOXO1-overexpressing cell line

U251 cells in the logarithmic growth stage were inoculated into 24-well plates. When the cell density reached 70-80%, one well was selected to be treated with the virus concentrate carrying the FOXO1-TurboID plasmid, and another well was selected to be treated with the virus concentrate carrying the pLenti-CMV-EGFP plasmid. Infection was carried out for 4–8 h, and the medium was replaced with fresh medium after infection. After 48 h, the fluorescence intensity of the cells infected with the pLenti-CMV-EGFP plasmid was observed. Intense fluorescence indicated that infection was successful. Next, the medium was replaced with medium containing puromycin (1.5 µg/mL) for resistance screening, and the cell state was observed within 24–48 h. After that, the surviving cells were further cultured, subcultured in 12-well plates when they were in a normal state, and then subcultured successively until the number of cells met the requirements of subsequent experiments. During this period, the cell state was observed every day, and the solution was changed every two days. Some cells were used to make a lysate for SDS‒PAGE. The cell line stably transfected with the pLenti-CMV-EGFP plasmid was named EGFP-U251, and the cell line stably transfected with the FOXO1-TurboID plasmid was named FOXO1-U251.

### Screening the time for biotin labeling

FOXO1-U251 cells were grown in 6-well plates, and when their density reached more than 80%, the original medium was removed, and the cells were washed twice with PBS. Biotin labeling was carried out with biotin (500 µmol·L^-1^), MgCl_2_ (1 µmol·L^-1^) and Adenosine 5’-triphosphate (ATP, 200 µmol·L^-1^), and six time points (0 min, 10 min, 1 h, 3 h, 6 and 12 h) were evaluated. At each time point, the cells were collected, and the cell lysates were subjected to SDS‒PAGE. The antibody used was streptavidin-peroxidase.

### Proximity labeling technology based on TurboID

According to the method reported by Branon et al.[22], EGFP-U251 and FOXO1-U251 cells were cultured in T75 cell culture flasks. The experiment was divided into three groups. ① In the blank group, EGFP-U251 cells were cultured in two T75 culture flasks; ② in experimental Group 1, FOXO1-U251 cells were cultured in two T75 culture flasks; and ③ in experimental Group 2, FOXO1-U251 cells were cultured in two T75 culture flasks and LPS (100 ng·L^-1^) was also used. All three groups of cells were treated with biotin (500 µmol·L^-1^), MgCl_2_ (1 µmol·L^-1^) and ATP (200 µmol·L^-1^) in DMEM, and the cells were collected for sample preparation after 12 h.

### Affinity purification of the biotin-labeled protein

The cells in each of the T75 culture flasks were collected in a 1.5 mL EP tube and resuspended by adding 1 mL of RIPA buffer. Then, the cells were lysed on ice for 15 min and centrifuged at 12,000 r·min^− 1^ for 10 min. After that, the supernatant was transferred to a clean 1.5 mL EP tube. Magnetic Dynabeads were washed with 1 mL of RIPA buffer for 2 min and placed on a magnetic stand for approximately 10 s of adsorption until the liquid became clear. This procedure was repeated five times after discarding the RIPA buffer. Following the last wash, the beads were divided into 3 aliquots, and the buffer was discarded when the beads precipitated. The above protein lysates were added to the prepared beads, sealed with parafilm, and shaken slowly at 4 °C in a 360-degree shaker overnight. After overnight incubation, the supernatant was discarded, and the beads were washed with 1 mL of RIPA buffer for approximately 1.5 min. This procedure was repeated once. The beads were then washed once with 1 mL of KCl (1 mol·L^− 1^), three times with Buffer 1 (100 mL of Buffer 1: 67 mL of NaCl (3 mol·L^− 1^), 1 mL of Tris-HCl (pH 7.4, 1 mol·L^− 1^), and 32 mL of H_2_O), and once with Na_2_CO_3_ (0.1 mol·L^− 1^). Then, the beads were washed once with 10% SDS for 2 min (or up to 3 min). After adding RIPA buffer, the beads were placed in a metal bath at 50 °C for 1 min. Then, the beads were washed twice with 1 mL of RIPA buffer, and 1 mL of RIPA buffer was added back. One-third of the samples were used for identification by silver staining, and the remaining samples were washed twice with PBS after discarding the RIPA supernatant and finally stored at -20 °C after removal of the liquid.

### Identification of the protein with biotin affinity by silver staining

One-third of the protein sample mentioned in the previous step was used for preparing a 10% SDS‒PAGE gel (1 mm thick, 10-well comb). Five microliters of 10× loading buffer was added to 30 µL of sample, and each sample (10 µL) was loaded after denaturation. The gel after electrophoresis was put into 100 mL of fixative solution (50 mL of ethanol + 10 mL of glacial acetic acid + 40 mL of deionized water) and placed on a shaker at 60–70 r·min^− 1^ for 20 min. After discarding the fixative solution, the gel was put into 100 mL of 30% ethanol (30 mL of ethanol + 70 mL of deionized water) and shaken gently on a shaker for 10 min. After discarding the ethanol, 200 mL of deionized water was added, and the sample was shaken gently on a shaker for 10 min. After discarding the water, 100 mL of sensitizing solution (1×) (1 mL of sensitizing solution (100×) + 99 mL of deionized water; used within 2 h of preparation) was added, and the sample was shaken gently on a shaker for 4–5 min. After discarding the sensitizing solution, 200 mL of deionized water was added, and the sample was shaken gently on a shaker for 1 min; this procedure was repeated once. After discarding the water, 100 mL of silver staining solution (1×) (1 mL of silver staining solution (100×) + 99 mL of deionized water; used within 2 h of preparation) was added, and the sample was shaken gently on a shaker for 30–40 min. After discarding the silver staining solution, 100 mL of deionized water was added, and the sample was shaken on a shaker for 0.5 min; this procedure was repeated once. After discarding the water, 100 mL of chromogenic solution (0.05 mL of chromogenic solution A + 20 mL of chromogenic solution B (5×) + 80 mL of deionized water; used within 20 min of preparation) was added for gentle shaking on a shaker for 3–10 min until a band appeared. After discarding the chromogenic solution, 100 mL of stop solution (5 mL of glacial acetic acid + 95 mL of deionized water) was added, and the sample was shaken on a shaker for 10 min. After discarding the stop solution, 100 mL of deionized water was added, and the sample was shaken on a shaker for 0.5 min; this procedure was repeated once. Pictures of the bands were taken and saved.

### Identification by label-free quantitative protein mass spectrometry

After identification by silver staining, the protein samples were sent to a company for label-free quantitative protein mass spectrometry analysis. The specific steps in this procedure were as follows.

#### Sample preparation

The bead samples obtained from the immunoprecipitation experiment were washed three times with precooled PBS to remove the remaining detergent. Then, bead samples were incubated in reaction buffer (1% SDC; 100 mM Tris-HCl, pH 8.5; 10 mM TCEP; 40 mM CAA) at 95 °C for 10 min for protein denaturation, cysteine reduction and alkylation. The eluates were diluted with an equal volume of H2O and subjected to trypsin digestion overnight at 37 °C by adding 1 µg of trypsin. The peptide was purified using homemade SDB desalting columns. The eluate was vacuum dried and stored at -20 °C for later use.

#### LC‒MS/MS detection

LC‒MS/MS data acquisition was carried out on Orbitrap Exploris 480 mass spectrometer coupled with an EASY-nLC 1200 system (both from Thermo Scientific) [43]. Peptides were picked up by an autosampler and transferred to a C18 analytical column (75 μm × 25 cm, 1.9 μm particle size, 100 Å pore size, Thermo) for separation. Mobile phase A (0.1% formic acid) and mobile phase B (80% ACN, 0.1% formic acid) were used for the 60 min gradient separation procedure. A constant flow rate of 300 nL/min was used. For DDA mode analysis, each cycle consisted of the acquisition of one full scan mass spectrum (R = 60 K, AGC = 300%, max IT = 20 ms, scan range = 350–1500 m/z) followed by 20 MS/MS events (R = 15 K, AGC = 100%, max IT = auto, cycle time = 2 s). The HCD collision energy was set to 30. The isolation window for precursor selection was set to 1.6 Da. The former target ion exclusion was set to 35 s.

#### Data analysis

Raw MS data were analyzed with MaxQuant (V1.6.6) using the Andromeda database search [44]. Spectral files were searched against the [45] Human proteome database using the following parameters: LFQ mode was checked for quantification; variable modifications, oxidation (M) & acetyl (protein N-term); fixed modification, carbamidomethyl (C); and digestion, trypsin/P. Matching between runs was used for identification transfer. The search results were filtered with a 1% FDR.

### FOXO1 immunoprecipitation (IP) experiment

The anti-Flag affinity gel was fully resuspended to form a homogeneous solution. Fifty microliters of the mixture (25 µL of gel) was placed into a new centrifuge tube for centrifugation at 8,000 r·min^− 1^ for 30 s. The gel was allowed to precipitate to the bottom of the centrifuge tube and then stand for 1–2 min before adding the sample. After removing the supernatant, 500 µL of TBS was added, and the gel was gently resuspended and centrifuged at 10,000 r·min^− 1^ for 30 s. Then, the supernatant was discarded. This procedure was repeated once. A certain amount of cell lysate was added, and the final volume was adjusted to 1 mL. The cell lysate was incubated slowly overnight at 4 °C. On the next day, the cell lysate was centrifuged (8,000 r·min^− 1^ for 30 s), the supernatant was removed, and the pellet was shaken with 0.5 mL of TBS before being centrifuged again (8,000 r·min^− 1^ for 30 s). All of the supernatant was removed, and the procedure was repeated three times.

### Immunofluorescence of FOXO1-overexpressing cells

FOXO1-overexpressing cells were subcultured in a 3.5 cm confocal dish to observe their growth state. When the cell density was above 80%, the medium was removed, and the cells were fixed with 4% paraformaldehyde for 15 min. Then, the cells were treated with 0.5% Triton X-100 at room temperature for 20 min, blocked with 5% normal goat serum for 30 min, and incubated with FOXO1 primary antibody at 4 °C overnight. Subsequent steps were carried out in the dark. The secondary antibody was added to the cells for incubation at room temperature for 1 h, and PBST was used to wash the cells 3 times. Next, DAPI was added dropwise, the cells were incubated in the dark for 5 min, and the nuclei were counterstained. PBST was used to wash the cells 4 times. After the addition of fluorescence quencher, the cells were photographed by confocal microscopy and stored.

## Electronic supplementary material

Below is the link to the electronic supplementary material.

Additional file 1

Additional file 2

## Data Availability

The label-free protein profiling data in this paper are stored in iProX with accession number PXD036171. The data access connection in ProteomeXchange is http://proteomecentral.proteomexchange.org/cgi/GetDataset?ID=PXD036171. The iProX data access link is https://www.iprox.cn/page/project.html?id=IPX0004891000. All other data and materials used to support the findings of this study are available from the corresponding author upon request.

## Abbreviations

PPIs: Protein-protein interactions
LPS: Lipopolysaccharide
DAPI, 4': 6-diaminyl-2-phenylindoles
FITC: Fluorescein isothiocyanate
cy3: Sulfo-Cyanine3
RIPA: Radio Immunoprecipitation Assay
ATP: Adenosine 5’-triphosphate
SDC: Sodium deoxycholate
TCEP: Tris (2-carboxyethyl) phosphine
CAA: 2-Chloroacetamide
LC-MS: Liquid Chromatography-Mass Spectrometry
CAN: Acetonitrile
DDA: Data dependent analysis
HCD: Higher energy collisional dissociation
LFQ: Label free quantification
FDR: False discovery rate
