# Supplementary material for: Study of FOXO1-interacting proteins using TurboID-based proximity labeling technology
Source: BMC Genomics. 2023 Mar 24;24:146. doi: 10.1186/s12864-023-09238-z (PMC10039511; doi:10.1186/s12864-023-09238-z)
Supplement: Supplementary file 2 — Additional file 2 [file 12864_2023_9238_MOESM2_ESM.pptx]

## Slide 1
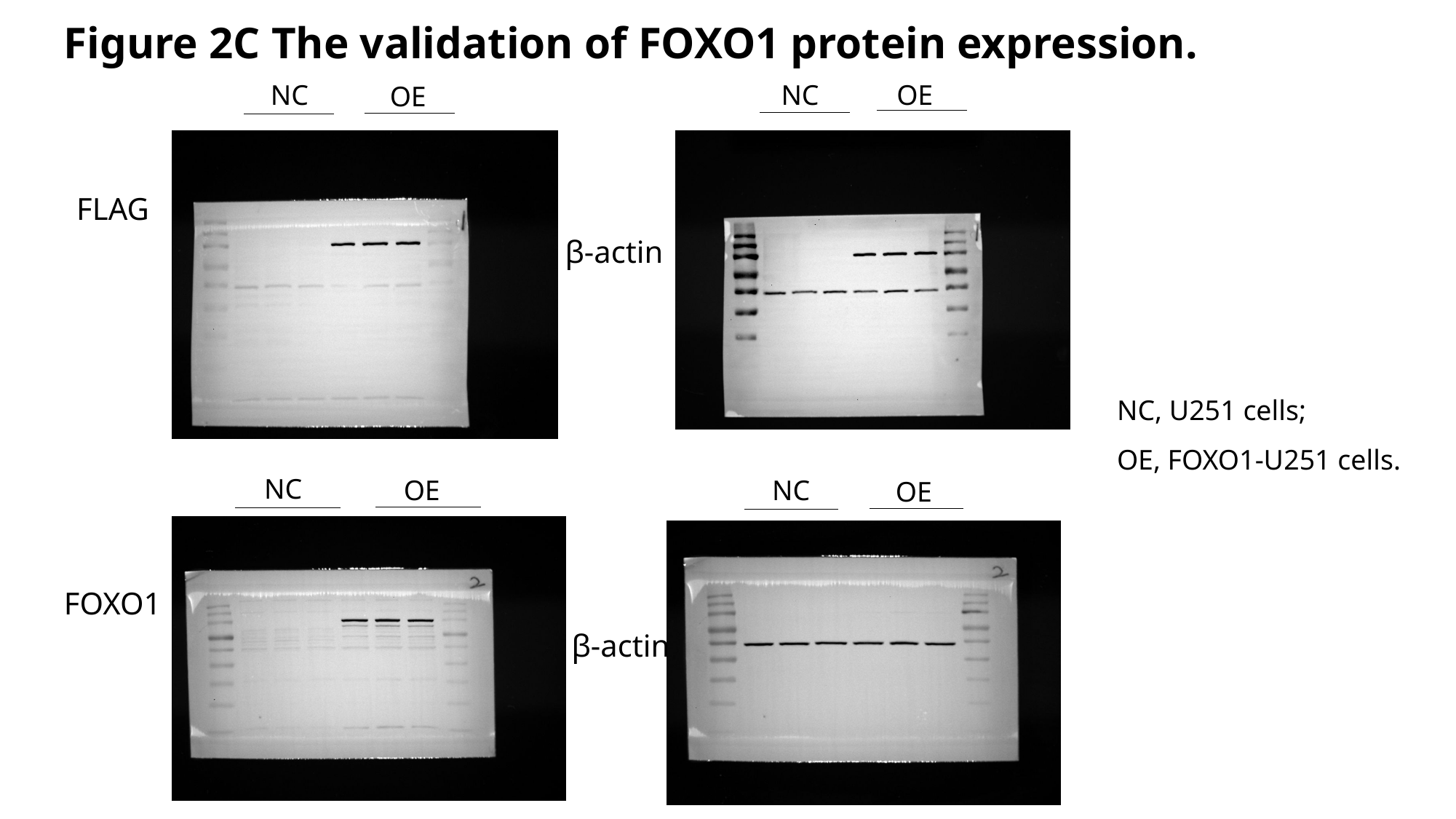

Figure 2C The validation of FOXO1 protein expression.
NC
OE
NC
OE
FLAG
β-actin
NC, U251 cells;
OE, FOXO1-U251 cells.
NC
OE
NC
OE
FOXO1
β-actin

## Slide 2
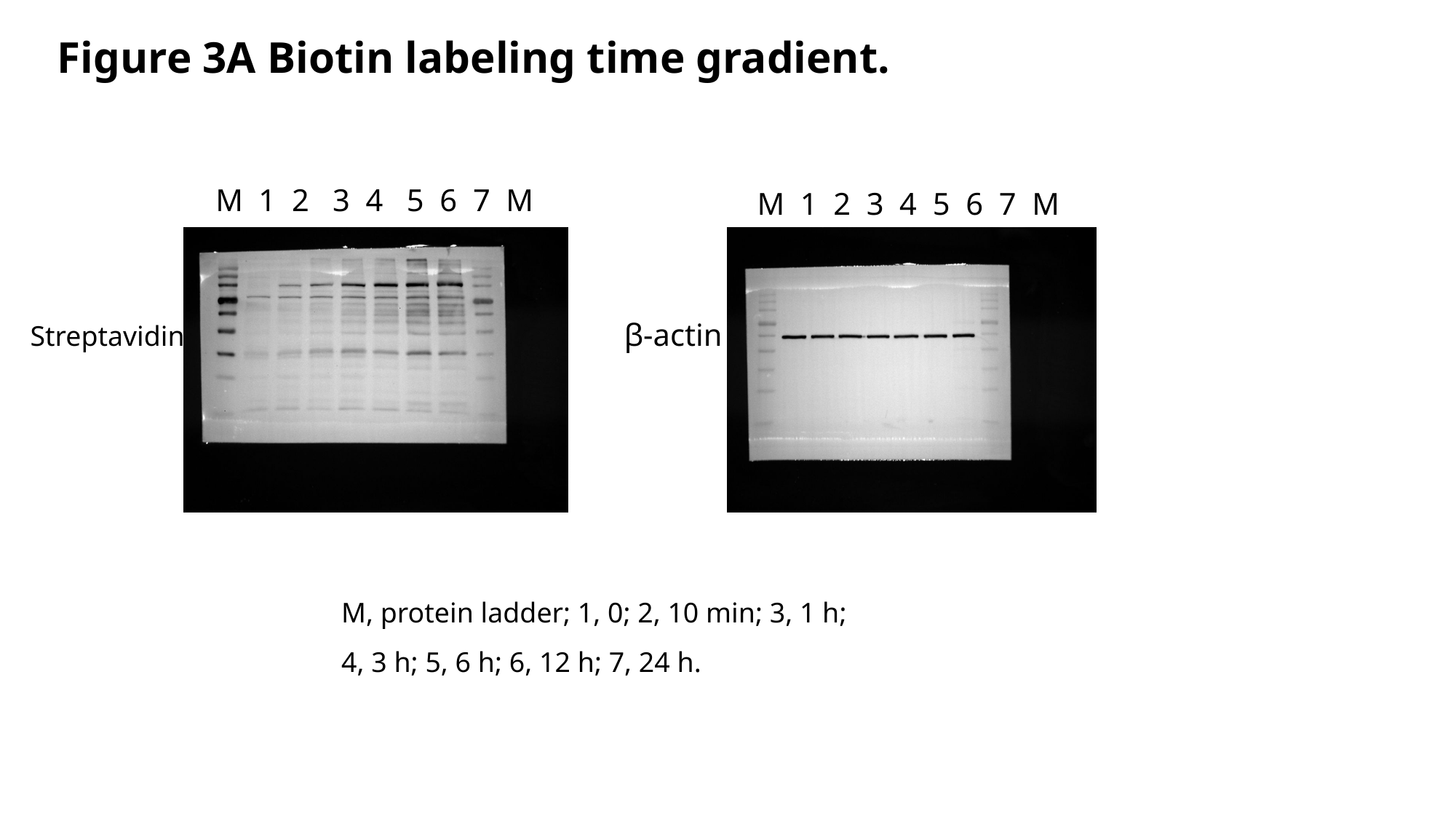

Figure 3A Biotin labeling time gradient.
M 1 2 3 4 5 6 7 M
M 1 2 3 4 5 6 7 M
β-actin
Streptavidin
M, protein ladder; 1, 0; 2, 10 min; 3, 1 h; 4, 3 h; 5, 6 h; 6, 12 h; 7, 24 h.

## Slide 3
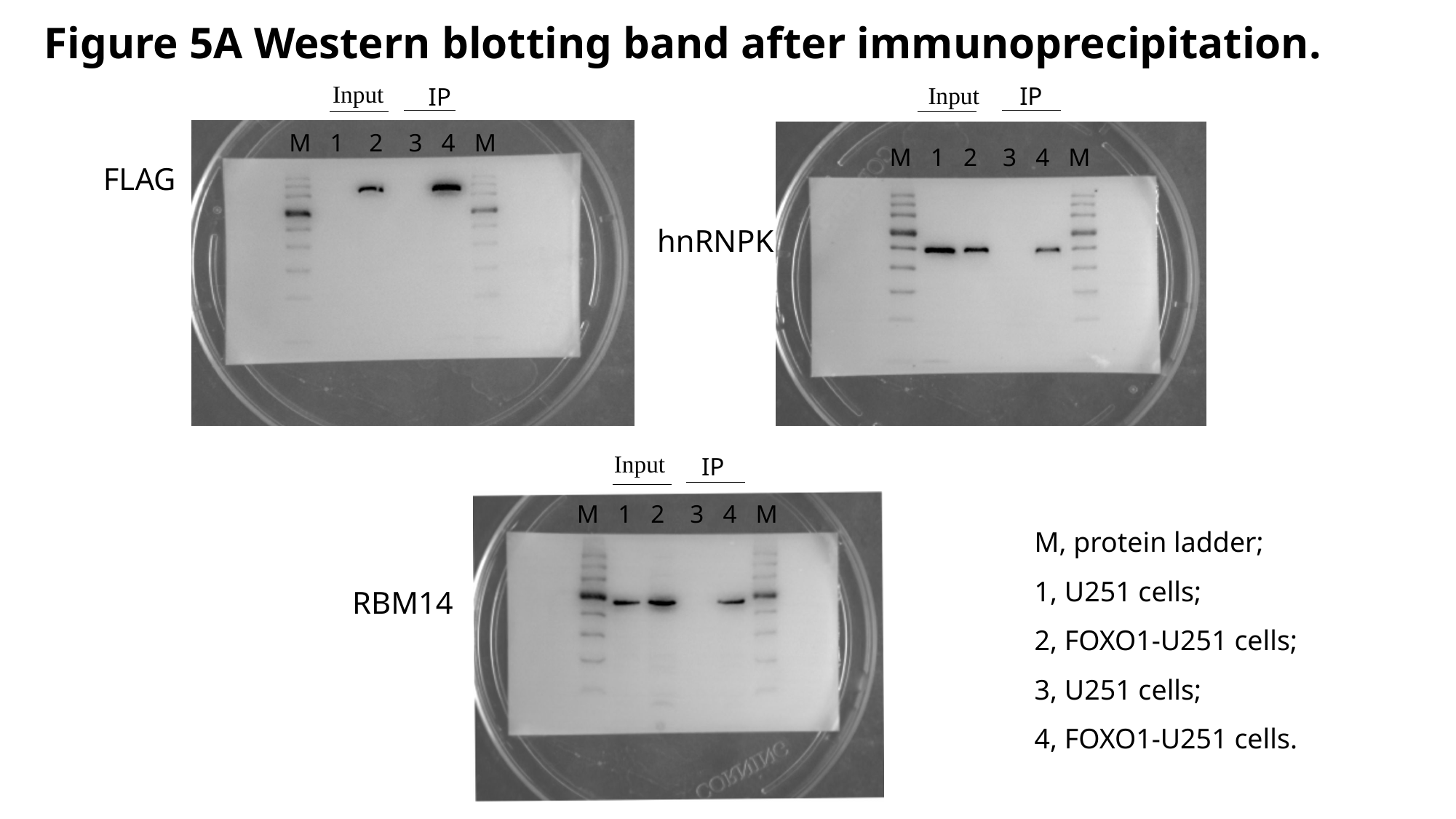

Figure 5A Western blotting band after immunoprecipitation.
Input
Input
IP
IP
 M 1 2 3 4 M
 M 1 2 3 4 M
FLAG
hnRNPK
Input
IP
 M 1 2 3 4 M
M, protein ladder;
1, U251 cells;
2, FOXO1-U251 cells;
3, U251 cells;
4, FOXO1-U251 cells.
RBM14
